# History and Direction of Gerontology and Caregiving

**DOI:** 10.1093/geroni/igaf122.932

**Published:** 2025-12-31

**Authors:** Steven Zarit

**Affiliations:** The Pennsylvania State University, University Park, Pennsylvania, United States

## Abstract

There has been so much research on caregiving in recent years that we do not recognize that it once was a backwater in the field. My goals are to consider where we have gotten, what we should emphasize, and next steps: (1) What is the patient’s disorder? The impact on patient and caregiver can be very different for different illnesses. (2) Most studies only look at caregivers. Karen Lyons has emphasized that we need to look at caregiver and patient as a dyad and analyze their findings together to understand benefits and potential harms. (3) Lyons’ also addresses another issue often overlooked in interventions, using measures that can potentially show change. A common example is using depression as an outcome but many of the caregivers in studies may not be depressed at the outset and so can’t show improvement. (4) Another issue is the family network and how that affects caregiver and patient. Family dynamics and support have a major effect on the primary caregiver. Bringing together family members in a therapeutic model has been one of the more promising interventions. (5) The caregiver’s social network. Support groups have become less common, but they can have considerable benefit in sharing ideas, learning skills, and learning about community resources. They may be effective but the early studies including my own may have used the wrong outcomes. (6) For group and family interventions, the leaders should have clinical training. There are excellent models of intervention that could achieve positive outcomes.

